# The Role of Catechins in Regulating Diabetes: An Update Review

**DOI:** 10.3390/nu14214681

**Published:** 2022-11-04

**Authors:** Lianghua Wen, Dan Wu, Xindong Tan, Meiqi Zhong, Jiabao Xing, Wei Li, Dan Li, Fanrong Cao

**Affiliations:** College of Horticulture, South China Agricultural University, Guangzhou 510640, China

**Keywords:** tea, hyperglycemia, insulin resistance, oxidative stress, mitochondrial damage, gut

## Abstract

Catechins are key functional components in tea and have many health benefits, including relieving diabetes. Glucose is necessary for maintaining life. However, when the glucose in the serum exceeds the threshold, it will lead to hyperglycemia. Hyperglycemia is mainly caused by insufficient insulin secretion or insulin resistance. Persistent hyperglycemia can cause various disorders, including retinopathy, nephropathy, neurodegenerative diseases, cardiovascular disease, and diabetes. In this paper, we summarize the research on the underlying mechanisms of catechins in regulating diabetes and elaborate on the mechanisms of catechins in alleviating hyperglycemia by improving insulin resistance, alleviating oxidative stress, regulating mitochondrial function, alleviating endoplasmic reticulum stress, producing anti-inflammatory effects, reducing blood sugar source, and regulating intestinal function. This review will provide scientific direction for future research on catechin alleviating diabetes.

## 1. Introduction

Diabetes is one of the most common metabolic diseases worldwide. According to its pathogenesis, it can be divided into diabetes mellitus (DM) type 1 (T1D) caused by insufficient insulin secretion and DM type 2 (T2D) caused by insulin resistance; both can lead to hyperglycemia in humans [1]. There are nearly 425 million diabetic patients worldwide (90% T2D), which is expected to increase to 700 million by 2045 [2]. Chronic hyperglycemia is connected to organ damage and failure, including eyes, kidneys, nerves, heart, gastrointestinal tract, and blood vessels. Meanwhile, multiple studies have demonstrated that diabetes, particularly T2D, can result in diabetic nephropathy, neuropathy, and cardiovascular and cerebrovascular illness [1]. Currently, pharmacological therapies such as insulin and metformin are primarily used to treat diabetes, but these therapies significantly raise the risk of cardiovascular disease and all-cause mortality [3]. Therefore, developing safe, non-toxic, and economically valuable functional food and/or nutritional medicines for diabetes replacement therapy is essential.

Tea is a globally popular beverage with rich bioactivities and health benefits. Catechins account for about 30% of the ingredients in finished tea; they possess antioxidant, anti-obesity, anti-tumor, anti-diabetic, and anti-inflammatory properties [4]. Studies have shown that catechins extracted from Indian medicinal plants can protect animals from alloxan-induced diabetes, possibly due to their free radical scavenging effect [5]. Additionally, catechins can alleviate DM via insulin resistance, alleviating oxidative stress, regulating mitochondrial function, alleviating endoplasmic reticulum (ER) stress, generating anti-inflammatory activity, lowering blood sugar sources, and regulating intestinal function [6,7,8]. In this paper, we comprehensively summarized the potential mechanisms of catechins in regulating diabetes in order to provide scientific guidance for further research on catechins alleviating diabetes.

## 2. Material and Methods

In this paper, authoritative literature search databases PubMed, Web of Science, and Google Scholar were used for comprehensive data collection (from the beginning of the database to October 2022), and key words related to catechin and diabetes were used for article searches, including “catechins”, “diabetes”, “EGCG”, “tea”, “tea and diseases”, “catechins and diseases”, “mechanism and diabetes”, “catechins and diabetes”, “EGCG and diabetes”, “catechins, insulin resistance and diabetes”, “catechins, endoplasmic reticulum stress and diabetes”, “catechins, inflammation and diabetes”, “catechins, intestinal function and diabetes”, and “catechins, oxidative stress and diabetes”. A manual article search was conducted to ensure that as many relevant studies as possible were obtained. The following inclusion and exclusion criteria were used in selecting articles:

Inclusion criteria:

- Articles written in English
- Cell, animal, and clinical studies
- Intervention with catechin supplementation

Exclusion criteria:

- Catechin supplementation in combination with other interventions (drugs/nutrients/exercise)
- The study does not clearly state the mechanism of diabetes relief

**Figure 1:**
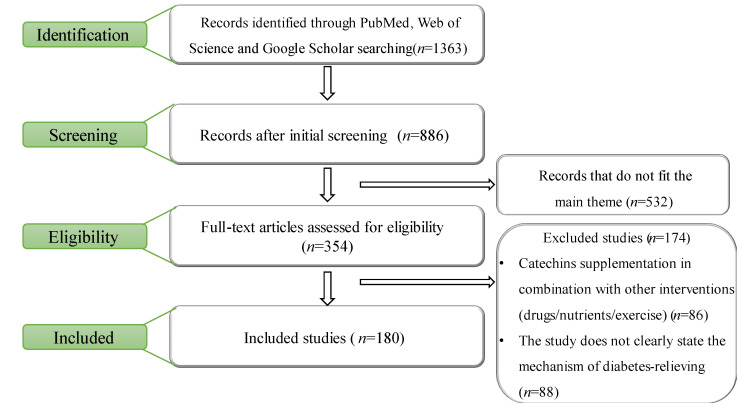
Study selection flow adapted from a systematic review by Muhammad Subhan Alfaqih et al. [9].

Data were extracted from the search results according to these standards, and the extracted data included: title, publication year, author, study subject, study objective, and catechin- and/or diabetes-related pathway of action. An initial search of the database yielded 1363 records. After reducing duplication and screening by title and abstract, an additional 354 full-text articles were analyzed. A total of 174 articles were excluded while 180 articles were used in this systematic review. A flow chart illustrating our study selection is shown in Figure 1.

Microsoft Office Professional Enhanced edition 2019 was used to collate and analyze the data, and ChemDraw 20.0 was used to draw the monomer chemical structure of catechins. Finally, all references in the paper are sorted and edited through Endnote 20.

## 3. Results and Discussion

### 3.1. Physical and Pharmacological Properties of Catechins

**Figure 2:**
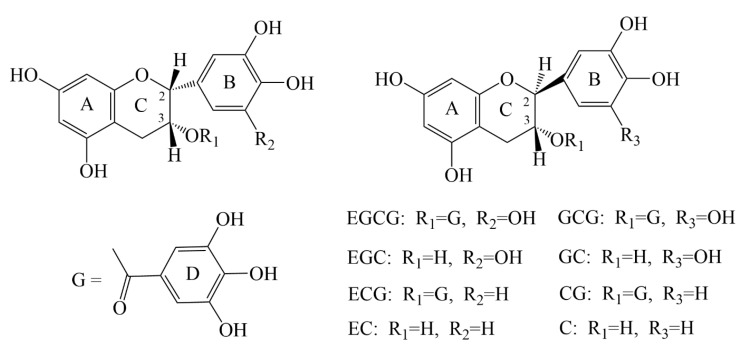
Chemical structures of eight catechin monomers.

Catechins are natural flavonoid polyphenols abundant in tea, broad beans, and grapes [10]. Catechins have two benzene rings (A and B rings) and a dihydropyran heterocycle ring (C ring) with a hydroxyl group at C3 carbon. This hydroxyl group can be esterified with a gallate group to form gallic conjugates, including (−)-epicatechin gallate (ECG), (+)-gallocatechin gallate (GCG), (+)-catechin gallate ester (CG), (−)-epigallocatechin gallate (EGCG) [11,12,13], (−)-epicatechin (EC), (+)-gallocatechin (GC), (+)-catechin (C), and (−)-epigallocatechin (EGC) [11,12,14] (Figure 2). Among these, EGCG accounts for 50–80% of the total catechins in green tea and has significant antioxidant, anti-inflammatory, anti-cancer, and anti-neurodegenerative properties [13,15].

**Figure 3:**
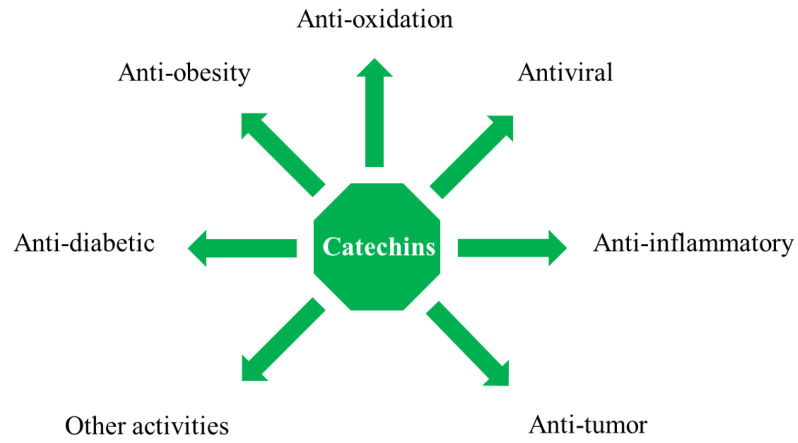
Pharmacological properties of catechins.

In recent years, research on catechins relieving diabetes has become more and more in-depth (Table 1). Research has shown that catechins isolated from Elaeagnus umbellate inhibit carbohydrate digestive enzymes, including α-amylase and α-glucosidase, and decrease fasting blood glucose levels in diabetic rats [16]. Chen et al. demonstrated that adipose-derived stem cells, incubated with EGCG in vitro, increased cell viability under high-glucose stress, and EGCG exhibited regeneration effects on damaged pancreatic tissues to alleviate diabetes [17]. A randomized placebo-controlled study indicated that participants' fasting plasma glucose significantly decreased after three months of daily enriched bread consumption [18]. In addition to their anti-diabetic pharmacological effects, catechins also have antioxidant [19], anti-tumor [20], anti-inflammatory [21], anti-microbial, anti-viral [22], anti-diabetic, anti-obesity, hypotensive [23], and cardiovascular disease prevention [24] properties (Figure 3).

### 3.2. Catechin Regulation of Diabetes

#### 3.2.1. Catechins Regulate Diabetes by Improving Insulin Resistance

**Figure 4:**
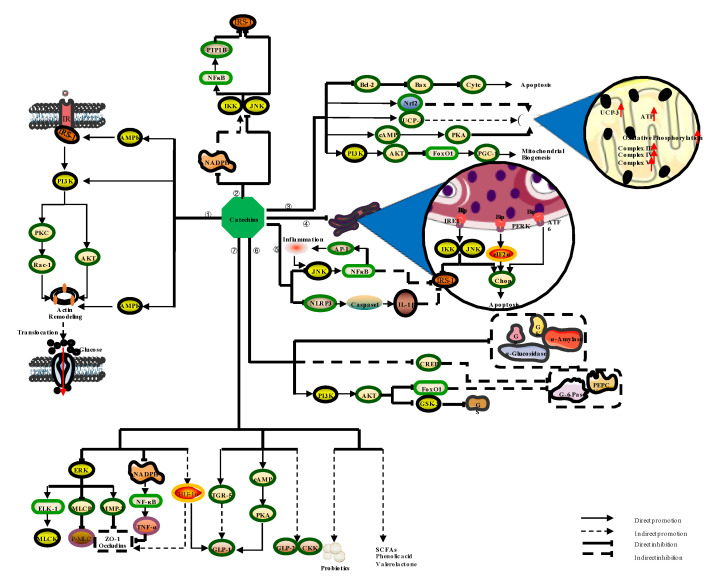
The pathway by which catechins regulate diabetes: (1) Catechins activate APMK and PI3K, and enhance insulin sensitivity, ultimately promoting uptake of glucose by the cells. (2) Catechins attenuate the inhibitory effect of NFκB on IRS-1 by suppressing the activity of NADPH, redox-sensitive signaling molecules IKK, JKN. (3) Catechins promote glucose-stimulated insulin secretion by increasing mitochondrial oxidative phosphorylation and ATP production, while increasing mitochondrial biogenesis and reducing mitochondria-associated enzyme-induced apoptosis of β-cells. (4) Catechins reduce ER stress by alleviating UPR, which leads to IRS-1-related decreased insulin sensitivity and β-cell apoptosis. (5) Catechins alleviate IRS-1-related insulin sensitivity reduction caused by inflammation by inhibiting NLRP3 and JNK. (6) Catechins decrease the source of blood glucose by reducing the activities of related enzymes, such as GK, GP, G-6-Pase, PEPCK, α-amylase, and α-glucosidase, and improve the activity of GS in promoting glycogen synthesis. (7) Catechins regulate diabetes by improving the gut barrier, balancing gut microbes, and promoting the secretion of glucose-related gut hormones.

Insulin signal transduction (IST) is the primary mechanism for maintaining the body’s blood glucose homeostasis. Normal IST involves multiple enzymes and intermediate mediators that ultimately lead to glycogen synthesis in hepatocytes and glucose uptake by adipocytes and muscle cells [36] (Figure 4). Insulin-like growth factor (IGF)-1, IGF-2, and insulin can activate the insulin receptor (IR), a transmembrane tyrosine kinase [37]. Then, IR autophosphorylates recruit the insulin receptor substrate (IRS) and the SHC-transforming protein-1 (SHC-1), and bind to these ligands. Subsequently, downstream signaling cascades are triggered through insulin-dependent kinases (e.g., AMPK and glycogen synthase kinase (GSK)-3) and insulin-inducible kinases (e.g., PKC, AKT, mammalian target of rapamycin (mTOR), and extracellular regulated protein kinase (ERK)1/2) [38,39]. AKT is activated when IRS-1 binds to PI3K. Next, AKT stimulates the translocation of GLUT4 from vesicles to the cell membrane, promotes glucose uptake, and suppresses GSK-3 to promote glycogen synthesis, regulating the body's blood glucose level [40].

Insulin resistance is a reduced response of insulin-targeted tissues to high physiological insulin levels and contributes to various diseases such as metabolic syndrome, nonalcoholic fatty liver disease, atherosclerosis, and T2D [41]. EGCG can mediate liver kinase B1 (LKB1) to activate AMPK, inhibit IRS-1 (serine 307) phosphorylation, and improve insulin sensitivity [42,43]. Notably, EGCG has different effects on various tissues. For example, EGCG activates IR and AMPK in adipocytes and promotes phosphorylation of IR and IRS-1 in hepatocytes [44]. On the other hand, EGCG activates AMPK without changing the phosphorylation of IR or IRS-1 in humans [45]. These differences might be related to variations in EGCG affinity for target proteins in various organs [29]. In addition, catechins can compensate for the negative effects caused by insulin resistance by enhancing IST, which promotes glucose transport into cells via GLUT4 and glycogen synthesis via glycogen synthase (GS). Interestingly, EGCG acts as an insulin-mimetic compound and promotes the translocation of GLUT4 to the cell membrane through the PI3K/AKT signaling pathway and enhances the cellular uptake of glucose [7]. Consistent with Zhang et al., EGCG-stimulated GLUT4 translocation needs to be mediated by PI3K/AKT, and EGCG can also promote GLUT4 translocation through the AMPK signaling pathway [46]. Manabu et al. suggested that EGCG might have a non-insulin-mimicking functional mode of action to regulate GLUT4 translocation. Afterwards, EGCG activates PI3K, which did not activate phosphorylate AKT, indicating that EGCG exerts its effect without AKT participation during this process, and inhibitors of PKC can inhibit the stimulatory effect of EGCG on GLUT4. Moreover, EGCG increases Ras-related C3 Botulinum Toxin Substrate 1 (Rac-1) activity and actin remodeling downstream of PKC, suggesting that EGCG mediates PI3K/PKC/Rac-1 to stimulate GLUT4 translocation to the cell membrane and promote glucose transport 28. Additionally, a complex composed of three components—hawthorn polyphenols, D-chiro-inositol (DCI), and EGCG—has synergistic hypoglycemic effects mediated by PI3K/AKT/GSK-3 of activating GS in the liver of STZ- or HFD-induced mice, and ultimately relieving insulin resistance and lower blood glucose [47]. Therefore, catechins can significantly modulate diabetes by improving insulin resistance.

#### 3.2.2. Catechins Regulate Diabetes by Alleviating Oxidative Stress

Endogenous factors, such as oxidative stress, can disrupt insulin signaling, reduce insulin sensitivity, and lead to an increased risk of diabetes [48,49]. The IST activity is reduced when partial sites of IRS-1 (e.g., serine 307) are phosphorylated by IKK and JNK redox-sensitive kinases [50] (Figure 4). JNK-1 knockout (JNK1-/-) mice are resistant to diet-induced obesity and have improved insulin sensitivity [51]. Additionally, animals with IKK genetic abnormalities are protected from developing insulin resistance [52]. On the other hand, oxidative stress promotes the production of PTP1B, a tyrosine phosphatase that reverses the tyrosine residues of IRS-1 phosphorylation to prevent insulin signaling [53]. PTP1B knockout mice are less likely to develop insulin resistance and obesity, and PTP1B inhibition has been proposed as a possible therapy for insulin resistance [54,55]. Antioxidants, particularly α-lipoic acid, vitamin E, and vitamin C, greatly increase insulin sensitivity [56,57].

Catechin is a natural antioxidant that relieves oxidative stress through various pathways, such as scavenging free radicals, chelating reducing metal ions, and enhancing antioxidant enzyme activity [58,59,60]. The NADPH oxidase family is a major source of intracellular superoxide and hydrogen peroxide, inducing oxidative stress. Meanwhile, EC and (-)-Epicatechin metabolites (ECM) suppress NADPH oxidase activity, inhibiting IKK and JNK activation, and ultimately improving insulin sensitivity [30]. Ahmed et al. showed that the activation of insulin signaling members (IR, IRS-1, AKT, and ERK1/2) is impaired in insulin-resistant rats induced by high fructose. At the same time, the negative regulators of these insulin signaling members are upregulated, including IKK, JNK, and PTP1B. However, these changes can be reversed by EC supplementation [61]. Anthocyanins, flavonoids as catechins, significantly reduce oxidative stress and inhibit HFD-induced obesity by suppressing the overexpression of redox-sensitive signals IKK/Nuclear factor κ-B (NF-κB), JNK1/2 and PTP1B, and insulin resistance in mice [62]. Furthermore, oligonol, a low molecular weight polyphenol in lychee fruit, is associated with JNK in ameliorating STZ-induced diabetes in rats [63]. Altogether, these results indicate that catechins suppress the reduction of insulin sensitivity via redox-sensitive signaling, which has a regulatory influence on diabetes.

#### 3.2.3. Catechins Regulate Diabetes by Improving Mitochondrial Function

Mitochondria are the final site of the oxidation of carbohydrates, lipids, and amino acids in eukaryotes and the organism's energy conversion center. Mitochondrial dysfunction has been linked to metabolic disorders such as obesity and cardiovascular disease and might cause diabetes and insulin resistance [64,65]. Mitochondria dysfunction can lead to toxic ceramide and diacylglycerol deposition, along with oxidative stress, finally triggering insulin resistance [66,67,68]. Pancreatic β-cells maintain physiological blood glucose levels by secreting insulin. Insulin secretion of β-cells is extracellularly coupled to glucose. When β-cells sense an increase in extracellular glucose concentration, they can absorb glucose through GLUT2. Subsequently, triphosphate (ATP) is released after the oxidation of glucose in mitochondria, which promotes the opening of calcium channels and ultimately leads to insulin secretion [69]. Therefore, it is evident that oxidative phosphorylation and ATP generation in mitochondria play essential roles in insulin secretion [70]. The uncoupling Protein-3 (UCP-3) gene is expressed on pancreatic islets, regulates ATP synthesis, and promotes insulin secretion [71,72]. In addition, studies have demonstrated that mitochondrial dysfunction and its induced apoptosis suppress insulin secretion. The PGC-1α family, which is involved in mitochondrial biogenesis, has been found to suppress insulin secretion in both mice [73] and patients with T2D [74,75]. In addition, proteins released by mitochondria-induced β-cell apoptosis, such as B-cell lymphoma (Bcl)-2-associated X protein (Bax) and cytochrome, can affect insulin secretion [76,77]. Interestingly, the connecting link between insulin resistance and mitochondrial dysfunction might be the IRS/PI3K/ Forkhead box O1 (FoxO1) pathway [78].

As mentioned above, mitochondria are essential for β-cell function and viability, and glucose-stimulated insulin secretion (GSIS) depends on mitochondrial respiration via the electron transport chain [79]. Treatment of β-cells with cocoa flavanols rich in EC increases mitochondrial complex III-V, ATP, GSIS, and mitochondrial respiration, as well as upregulating nuclear factor erythroid 2-related factor (Nrf) 1, Nrf 2, and GA-binding protein transcription factor alpha subunit (GABPA) [80]. Moreover, EC might stimulate Nrf2 to dissociate from cytoplasmic Kelch-like ECH-associated protein 1 (KEAP1) and translocate into the nucleus, thereby activating downstream target gene transcription, increasing the expression of mitochondrial complex III-V, enhancing mitochondrial respiration, and ultimately increasing intracellular ATP and GSIS. What is more, APT production and oxidative phosphorylation are related to UCP-3. EGCG can also stabilize mitochondrial function by increasing the UCP-3 expression, maintaining ATP synthesis and mitochondrial membrane potential, reducing β-cell apoptosis, and enhancing cell activity and insulin secretion [81]. As previously mentioned, FoxO1 is the bridge connecting insulin resistance and mitochondrial dysfunction. Studies have shown that EGCG supplementation successfully ameliorates diabetes-related mitochondrial deficiency and dysfunction, possibly due to the reduction in Foxo-mediated autophagy [82]. Mitochondrial function is also inseparable from mitochondrial biogenesis. Resveratrol, a plant polyphenol, restores STZ-induced PGC-1, mitochondrial transcription factor A (mtTFA), and Nrf1 in diabetic mice via mitochondrial biogenesis-related proteins [83]. Apoptosis of β-cells is an important cause of decreased insulin secretion. The Bcl-2 protein family regulates cytochrome c release as a vital step of apoptosis promotion. Bcl-2 inhibits the pro-apoptotic enzyme Bax to prevent mitochondrial cytochrome c release. EGCG increases Bcl-2 expression to protect cells against TNF-α, Interferon-gamma (IFN-γ), and Interleukin (IL)-1β-induced apoptosis via the mitochondrial pathway and restores GSIS [84]. Interestingly, deletion of the circadian-rhythm-related gene in the brain and muscle, the Arnt-like protein 1 (Bmal1), results in mitochondrial abnormalities and markedly attenuates oxidative phosphorylation [85]. EGCG can also activate PI3K/AKT and AMPK signaling pathways in a Bmal1-dependent manner to improve glucosamine-induced ROS, abnormal mitochondrial membrane potential, and downregulation of the mitochondrial respiratory complex, and alleviate insulin resistance [26]. Overall, catechins regulate diabetes by increasing mitochondrial function, primarily by sustaining oxidative phosphorylation and ATP generation, mitochondrial biogenesis, and mediating mitochondrial cell protection.

#### 3.2.4. Catechins Modulate Diabetes by Alleviating ER Stress

The ER is essential for protein synthesis and transport, and its dysfunction easily leads to chronic diseases such as obesity and diabetes [86]. Insulin is secreted by β-cells as a protein hormone that regulates diabetes. A single β-cell can produce 1 million insulin molecules every minute, accounting for about half of the total protein produced by β-cells [87,88]. Thus, β-cells must have a well-developed ER function to produce insulin in response to high blood glucose levels.

The T2D impairs ER homeostasis and leads to an accumulation of unfolded or misfolded proteins in the ER lumen, and activates the unfolded protein reaction (UPR) intracellular signaling [89]. The UPR is conducted by three proteins on the ER membrane: PKR-like ER-regulated kinase (PERK), inositol-requiring protein 1 (IRE1), and activating transcription factor (ATF) [6]. When UPR is activated, PERK activates ATF4, regulating the transcription of ER-associated protein degradation (ERAD) and autophagy genes [90]. At the same time, the activated IRE1 acts as an endonuclease to degrade mRNA around the ER and decrease the quantity of protein entering the ER. It also acts as a kinase to initiate JNK-mediated apoptosis [91,92]. Besides, the C/EBP-homologous protein (CHOP), regulated by ATF6, can induce autophagy, apoptosis, and insulin resistance [93,94].

At the animal level, EC supplementation downregulates PERK and IRE1, alleviates oxidative stress and tissue inflammation mediated by ER stress, and attenuates obesity-associated insulin resistance in adipose tissue from HFD-induced obese mice [95]. Another animal study used A-type dimeric EGCG to improve insulin resistance and elevated blood glucose in rats based on the inhibition of ER stress-induced apoptosis and G-6-Pase. Specifically, the A-type dimeric EGCG lowered the levels of ATF4, p-JNK, p-IRE1, and p-PERK [96]. Generally, binding immunoglobulin protein (BiP) is a crucial ER stress signal bound to ER transmembrane proteins and inhibits protein aggregation around the ER [97]. At the cell level, similar to the animal level, EGCG suppresses BiP expression and PERK phosphorylation, leading to ER stress and apoptosis and improving diabetic nephropathy [98]. Similar to catechins, other polyphenols can also participate in alleviating ER stress, including curcumin and tyrosol. Curcumin can diminish palmitate-induced insulin resistance, inhibit ER stress/JNK/IRS-1 signaling in human umbilical vein endothelial cells, and maintain ER homeostasis by inducing autophagy and promoting the degradation of damaged and aggregated proteins [99]. Tyrosol can dose-dependently lower pancreatic β-cell apoptosis, which is connected to its inhibition of ER stress and reduced CHOP gene expression [100]. In summary, catechins can reduce ER stress to manage diabetes, primarily by alleviating UPR and its related apoptotic signaling.

#### 3.2.5. Catechins Regulate Diabetes via Anti-Inflammatory Effects

Studies have demonstrated the link between inflammation and diabetes [101]. Adipose tissue is a major source of inflammatory markers and a target of the inflammatory process in diabetes [102]. Research has demonstrated that (Figure 4) sustained nutritional stimulation causes adipocyte hypertrophy, followed by an increase in various pro-inflammatory cytokines and chemokines (e.g., TNF-α, IL-6, Monocyte chemoattractant protein (MCP)-1) [103,104,105,106,107], creating a chemotactic gradient that attracts more monocytes and other immune cells to accumulate and produce more cytokines and chemokines, finally exacerbating inflammation [108]. These pro-inflammatory factors activate intracellular signaling molecules, such as JNK, leading to nuclear translocation of NF-κB and activation of Activator protein-1 (AP-1), ultimately suppressing IRS-1 and inducing more inflammatory mediators [107,109]. Furthermore, an additional key component of inflammatory activation is the multimeric protein complex NLRP3, activated by cell nutrients, such as glucose and free fatty acids. NLRP3 regulates the activation of caspase-1 that cleaves precursor cytokines, such as Pro-IL-1β, resulting in increased IL-1β activity in tissues [110,111]. IL-1β inhibits IRS-1 expression at the transcriptional level through an ERK-dependent mechanism and increases the expression of Inducible Nitric Oxide Synthase (iNOS), resulting in β-cell destruction [112,113]. Some statins used in diabetes patients have been proven to reduce inflammatory markers by 61%. These results indicate, at least in part, that suppressing inflammation helps alleviate diabetes.

Currently, polyphenols such as resveratrol and luteolin have been shown to alleviate diabetes, and this effect is related to NF-κB signaling molecules [114,115]. Catechins are natural polyphenols that can alleviate diabetes. For example, Kim et al. found that in the β-cell line RINm5F and islets, 0.1–1 mM EC could inhibit IL-1β-induced nitrite production (a downstream product of NO) and promote insulin release by inhibiting the NF-κB pathway [113]. Additionally, EGCG can reduce cytokine-induced β-cell death by inhibiting NF-κB activation, which downregulates iNOS [116]. The production of cytokines and chemokines is crucial in developing diabetes after NF-κB activation [117]. Grape seed extract containing catechins, epicatechin, gallic acid, and proanthocyanidins can significantly decrease HFD-induced plasma levels of TNF-α, IL-6, and MCP-1 in obese mice and improve macrophage infiltration in liver tissue and insulin sensitivity [118]. NLRP3, another key component of inflammatory activation in diabetes, is also involved in regulating catechins in diabetes. Zhang et al. showed that EGCG improved HFD-induced glucose tolerance deterioration and T2D through the direct inhibitory impact on NLRP3, suppressing caspase-1 activation and IL-1β production [32]. These studies imply that catechins regulate NF-κB and NLPR3-related inflammatory signal molecule activation to ameliorate β-cell damage and insulin sensitivity caused by cytokines and chemokines.

#### 3.2.6. Catechins Regulate Diabetes by Inhibiting the Source of Blood Glucose

The T2D is characterized by hyperglycemia, which can lead to various diseases, including cardiovascular disease, hypertension, and retinopathy. Therefore, regulating glucose production is effective in controlling blood glucose levels. The digestion and absorption of carbohydrates is the most common way of increasing blood glucose, which requires the participation of enzymes containing α-amylase and α-glucosidase. Thus, inhibitors of these enzymes are commonly used to treat diabetes [7]. Gluconeogenesis, another type of glucose production, is the reverse route of glycolysis and involves several enzymes, especially PEPCK, G-6-Pase, and Fructose 1,6-bisphosphatase (FBP) [119]. PEPCK and G-6-Pase are regulated by FoxO1, a transcription factor suppressed by insulin/AKT signaling [120,121]. The Cyclic AMP response element binding protein (CREB) is another transcription factor that can induce PEPCK and G-6-Pase gene expression [122,123,124]. Therefore, metformin, which plays a hypoglycemic role by inhibiting gluconeogenesis, is usually used as the first-line drug in T2D patients [125,126]. Furthermore, in the early stage of fasting, the primary strategy to maintain glucose levels is glycogenolysis, in which the key enzyme is glycogen phosphorylase (GP) [127].

Diabetic patients have difficulties managing postprandial blood glucose levels generated by carbohydrate digestion and absorption on their own, and supplementation with α-glucosidase and α-amylase inhibitors is a typical way of suppressing the postprandial rise of blood glucose [86]. EGCG is the most effective α-glucosidase inhibitor among catechins because of its gallic acyl group and the hydroxyl structure on the B-ring [128]. Specifically, EGCG connects to α-glucosidase with hydrogen bonds and changes the secondary structure and microenvironment of α-glucosidase in a reversible and non-competitive way, ultimately inhibiting the activity of α-glucosidase [7]. Furthermore, EGCG also improves T2D by binding to the active site of α-amylase, which hydrolyzes α-1,4-glycosidic bonds of starch to produce monosaccharides [129]. Regarding the relationship between catechins, gluconeogenesis, and glycogenolysis, EGCG inhibits CREB activation and FoxO1 nuclear translocation, suppressing gluconeogenic gene expression [124]. Additionally, catechins isolated from cassia seeds can restore glucokinase (GK) G-6-Pase, GS, and GP to normal levels in STZ-induced diabetic rats [130]. Overall, the decrease of glucose sources by catechins to control blood glucose levels is a key pathway for DM regulation, and its fundamental mechanism is depicted in Figure 4.

#### 3.2.7. Catechins Regulate Diabetes by Improving Intestinal Function

The gut is directly related to human health, particularly in the development of metabolic diseases, such as obesity and T2D, and acts as the pathological core of metabolic syndromes via: (1) direct mutual contact with various diets and metabolites; (2) nutrient digestion and absorption, as well as energy homeostasis regulation; (3) an essential site for endotoxin synthesis and a gut barrier that prevents endotoxins from entering circulation; (4) the presence of a diverse microbial community; (5) regulation of gut hormones and satiety promotion, as well as glycolipid homeostasis [131,132,133].

Generally, pathogens and toxic substances are present in the gut, but there is also a defensive system, the gut barrier, to prevent them from accessing the organism's internal environment. The gut barrier is based on the production of many components, including intestinal epithelium, anti-microbial peptides, antibodies, and mucus [134]. The tight junction (TJ) is a protein complex that operates as a paracellular gut barrier, connecting neighboring cells and controlling paracellular substance transport. The TJ is also composed of transmembrane proteins (e.g., claudins and occludins) and peripherin (e.g., zonula occludens (ZO)-1, ZO-2, ZO-3) [135]. TJ opening requires phosphorylation of the myosin light chain (MLC), regulated by the phosphorylation of myosin light chain kinase (MLCK) and dephosphorylation of myosin light chain phosphatase (MLCP) [136]. When TJ is impaired, the subsequent increase in intestinal permeability leads to the entry of pathogens and endotoxins (e.g., lipopolysaccharide (LPS)) into circulation, causing tissue damage and a systemic inflammatory response, ultimately inducing insulin resistance [137]. Obesity and insulin resistance are not detected in germ-free mice given an HFD but are observed when microorganisms from HFD-induced obese animals are transplanted to germ-free mice [138]. This indicates that diabetes might be related to the microbiome. Additionally, hormones secreted by the gut, such as glucagon-like peptide (GLP)-1, GLP-2, and cholecystokinin (CKK), can regulate diabetes [139].

As mentioned above, increased intestinal permeability can raise the risk of diabetes, and EC can improve intestinal permeability induced by HFD in vivo. Meanwhile, EC can prevent Caco-2 monolayer permeabilization through ERK1/2 modulation, which can upregulate MLCP expression [136,140]. The activation of mitogen-activated protein kinase (MEK) can activate ERK, increasing the activity of matrix metalloproteinase (MMP)-2 and MMP-9, which degrade TJ-related proteins like ZO-1 and ocludins [141]. Wang et al. found that EC inhibits MMP-2 activity to suppress Caco-2 monolayer permeabilization, which might be related to ERK inhabitation. Similar to ERK, NF-κB is a key regulator of TJ structure and dynamics and is activated by NADPH [136]. EC-rich diet supplementation helps improve intestinal permeability because EC inhibits NADPH and NF-κB, and NF-κB and its induced TNF-α lead to downregulation of ZO-1 [142,143]. Moreover, catechin-rich green tea extract (GTE) is related to the hypoxia-inducible factor (HIF)-1α in downregulating TJ-related proteins induced by HFD [144]. Indeed, HIF-1α is involved in the regulation of TJ-related protein expression [145]. Dey et al. found a positive correlation between HIF-1α and claudin-1 expressions, but the regulation of TJ by GTE via HIF-1α remains unknown [144].

Microbes are considered the "second genome" of humans, responsible for more than 98% of the genetic activity of the organism [146]. Intestinal microbes play an important role in the development of diabetes [147]. According to previous reports, GTE and green tea promote the growth of Lactobacillus and Bifidobacterium, probiotics that help glucose levels [148,149,150]. At the phylum level, the ratio between Firmicutes and Bacteroidetes, a marker of intestinal imbalance, is reduced by EGCG [151,152]. Short-chain fatty acids (SCFAs) and catechin metabolites in the gut are beneficial for improving diabetes [153,154,155,156]. EGCG and GCG greatly enhance the production of SCFAs in vitro, improving intestinal barrier integrity via HIF-1α [157]. Another study showed that green tea promotes the growth of SCFA-producing microorganisms. Intestinal microorganisms are in direct contact with catechins, which might affect their biological activities. For example, green tea rich in catechins attenuates the function of microorganisms to metabolize amino and nucleotide sugars, providing the skeleton for LPS synthesis and helping reduce LPS-induced damage to the body and insulin resistance [144].

The intestine is not only the main site of digestion and absorption but also secretes some hormones, including GLP-1, GLP-2, and CKK, to regulate diabetes [139,158]. Specifically, GLP-1 improves glucose homeostasis and can be used to treat T2D [159,160]. GLP-2 enhances intestinal barrier integrity and regulates energy balance [161,162]. However, the deletion of the CKK gene exacerbates hyperglycemia [163]. EC can decrease DPP-IV activity in vivo, degrading pro-glucagon, increasing the expression of pro-glucagon, and producing GLP-1 and GLP-2 [164,165]. EC supplementation can also raise GLP-2 levels. In addition, EGCG increases CKK production in the duodenum and GLP-1 secretion in the ileum in vitro [166]. The major mechanisms of catechins regulate intestinal function by improving the intestinal barrier and microbial community, and boosting intestinal peptide production to treat diabetes (Figure 4).

## 4. Discussion

Catechins alleviate diabetes by improving insulin resistance, alleviating oxidative stress, regulating mitochondrial function, alleviating ER stress, producing anti-inflammatory effects, reducing blood sugar sources, and regulating intestinal function. Many signaling molecules, especially stress signaling molecules, are activated in diabetic patients. Hence, diabetes is not affected by a simple linear regulatory mechanism but by a complex regulatory network composed of many signaling molecules. As a typical stress signaling molecule, JNK regulates oxidative stress, ER stress, and inflammation for catechin to improve diabetes [61,96]. Moreover, catechins alleviate inflammation and oxidative stress by regulating JNK [96,167]. Thus, JNK might be the core of oxidative stress, ER stress, and inflammation. What is more, the complex regulatory network composed of these three elements might provide more therapeutic targets for catechins to improve diabetes. 

Because of their antioxidant activity, catechins, particularly EGCG, have a role in treating various diseases such as diabetes, obesity, and cancer [168]. The antioxidant activity of EGCG is related to its amount and place of phenolic hydroxyl groups [11]. However, an increasing number of in vitro and in vivo investigations have demonstrated that EGCG has pro-oxidant activity because of its instability and autoxidation [169,170]. Under normal physiological circumstances (pH 7.4, 37 °C), EGCG is autoxidized to o-quinone with the production of superoxide anion radicals, O2- and H_2_O_2_ [171], converting the trivalent ion Fe (III) to the divalent ion Fe (II), hastening the Fenton reaction, and producing OH^-^ [172,173]. This pro-oxidative effect of EGCG also has benefits, such as the inhibition of Escherichia coli and the promotion of apoptosis in cancer cells [174,175]. EGCG activates IR and might be beneficial for alleviating diabetes by increasing hydrogen peroxide levels, a reactive oxygen species [44]. In addition, although the improvement of free fatty acid-induced insulin resistance by EGCG is related to the enhancement of antioxidant enzyme expression, it has not been directly demonstrated that this improvement occurs by alleviating oxidative stress [176]. Since the antioxidant and pro-oxidant activities of EGCG can vary with concentration [177], its dose effect and underlying mechanisms in improving diabetes need to be elucidated by further studies.

As mentioned above, catechins can alleviate diabetes via multiple pathways. However, the low bioavailability of catechins has a certain impact on its health efficacy. Generally, the low bioavailability of tea polyphenols is attributed to poor gastrointestinal absorption, with less than 2% of the oral EGCG dosage being found in the blood of rats [178]. Therefore, improving the bioavailability of catechins is crucial to improving their pharmacological properties. Additionally, improving tea polyphenol stability in oxygen, acidic, and alkaline conditions can significantly boost bioavailability. Tea polyphenols have been modified using peracetic acid to preserve the free hydroxyl group around the molecule and increase stability [179]. What is more, using carriers or capsules as delivery systems not only minimizes the instability of tea components but also enhances solubility and increases permeability in the intestine, resulting in higher plasma concentrations and improving the bioavailability and biological activity [180]. Hence, resolving the issue of catechin bioavailability is critical to improve its diabetes-relieving effects.

## 5. Conclusions

In summary, catechins modulate DM via multiple pathways: (1) improving insulin resistance; (2) reducing oxidative stress; (3) regulating mitochondrial function; (4) reducing ER stress; (5) having anti-inflammatory effects; (6) lowering blood glucose sources; (7) modulating intestinal function. The use of catechins in managing diabetes remains contentious but most findings imply that catechins or catechin-rich diets, such as green tea intake, have various beneficial effects in DM patients. Therefore, catechins are potential multi-target treatment agents for DM, but require additional research in the future.

## Figures and Tables

**Table 1:** Improvement of diabetes through catechins.

Model	Material	Main Conclusion	Reference
L6 skeletal muscle cells	EGCG	EGCG inhibited α-glucosidase activity while increasing glucose transporter (GLUT)4 translocation to the membrane and glucose absorption via the Phosphatidylinositol-3 Kinase (PI3K)/ protein kinase B (AKT) pathway.	[7]
3T3-L1 preadipocyte cell	EGCG	By reducing oxidative stress and mitochondrial dysfunction, EGCG reduced fat production and accumulation, while also attenuating the tumor necrosis factor -α (TNF-α)-induced insulin signaling pathway blockage.	[25]
HepG2 cell	EGCG	By improving insulin signaling and reducing oxidative stress, EGCG modulated metabolic diseases related to the biological clock.	[26]
HepG2 cell	EGCG	Through the GLUT2/Peroxisome proliferator-activated -γ coactivator (PGC)-1β/sterol regulatory element-binding-1c (SREBP-1c)/ fatty acid synthase (FAS) pathway, EGCG reduced glucose and PA-induced inflammation, oxidative stress, and free fatty acids, ultimately reducing insulin resistance.	[27]
INS-1 cell line	EC	Physiological concentrations of EC promoted insulin secretion from saturated fatty acid-impaired beta cells by activating the Ca^2+^/calmodulin-dependent protein kinase (CaMK) Ⅱ pathway.	[28]
L6 myoblasts and ICR mice	EGCG	EGCG at physiological concentrations reduced postprandial glucose levels via insulin- and 5’-Adenosine monophosphate-activated protein kinase (AMPK)-dependent pathways in L6 cells, whereas it promoted GLUT4 translocation via PI3K and AMPK pathways in the ICR mouse flounder muscle.	[29]
HepG2 cell and high fat diet (HFD)-induced mice	EC and EC metabolites (ECM)	Palmitate induced increases in NADPH oxidases (NOX)3/NOX4 expression, upregulation of c-Jun N-terminal kinase (JNK) and IκB kinase (IKK) activities, and decreased insulin sensitivity were all inhibited by EC and ECM.	[30]
3T3-L1 adipocytes, RAW264.7 macrophages and HFD-induced macrophages and mice	EC	Chemokine ligand 19 (CCL19) downregulation by the EC improved adipose tissue inflammation while also inhibiting HFD-induced obesity and insulin resistance.	[31]
Mice with HFD-induced T2D	EGCG	EGCG improved glucose tolerance and alleviated Nod-like receptor protein (NLRP)3-dependent inflammation.	[32]
HFD and streptozotocin (STZ)-induced T2D in SD rat	EGCG	In the diabetic rat model, EGCG continued to improve glycemic control and insulin sensitivity while decreasing lipid profile and oxidative stress.	[33]
HFD- and STZ-induced T2D in ICR rat	EGCG	EGCG inhibited α-amylase and α-glucosidase activity, as well as Phosphoenolpyruvate carboxy kinase (PEPCK) and glucose-6-phosphatase (G-6-Pase) expression and gluconeogenesis.	[8]
HFD-induced insulin resistance in mice	EC	EC improved insulin sensitivity induced by HFD by downregulating JNK, IKK, protein kinase C (PKC), and protein tyrosine phosphatase 1B(PTP1B).	[34]
Mice and 39 healthy people	EGCG and Green tea	Catechin consumption in the evening was more effective at lowering postprandial blood glucose levels.	[35]

## Data Availability

Not applicable.
